# Effect of local ozone treatment on rats with anterior rectal resection and the possible mechanisms

**DOI:** 10.1186/s12938-021-00918-z

**Published:** 2021-08-06

**Authors:** Wei Zhang, Meng Wu, Peng Chen, Jiamin Zhang, Jiaze Ma, Yile Cheng, Xiaoliu Li, Junjie Hu, Wanli Li, Yuxin Du, Kang Ding, Zhimin Fan

**Affiliations:** 1 Department of Anesthesiology, Nanjing Hospital of Chinese Medicine Affiliated to Nanjing University of Chinese Medicine, Nanjing, 210012 Jiangsu China; 2 Nanjing University of Chinese Medicine, Nanjing, 210000 Jiangsu China; 3 National Center of Colorectal Surgery, Nanjing Hospital of Chinese Medicine Affiliated to Nanjing University of Chinese Medicine, No. 157 Daming Road, Qinhuai District, Nanjing, 210012 Jiangsu China

**Keywords:** Anterior rectal resection, Inflammation, Oxidative stress, 5-HT, TRPV

## Abstract

**Background:**

Anterior resection syndrome (ARS) is characterized by the diverse and interchangeable evacuatory symptoms that may occur following distal colorectal resection. We aimed to investigate the effect and potential mechanisms of ozone perfusion on rats with anterior rectal resection (ARR).

**Material and methods:**

After establishment of rat ARR model, 20, 40 and 80 ug/ml ozone was used to treat rats by enema administration. The pathological examination of intestinal tissue was detected using hematoxylin–eosin staining. The rate of loose stools, minimum threshold volume of abdominal withdrawal reflex (AWR) and Bristol grade were used to evaluate the degree of abnormal defecation function. Subsequently, the levels of oxidative stress- and inflammation-related markers, 5-hydroxytryptamine (5-HT), inducible nitric oxide synthase (iNOS) and nitric oxide (NO) in the serum and intestinal tissue were determined with the corresponding kits. Meanwhile, the expression of nuclear factor kappa B (NF-κB) p65, transient receptor potential vanilloid (TRPV)1, TRPV4, iNOS and 5-HT receptor 3A (5-HTR3A) was determined with RT-qPCR and western blotting.

**Results:**

Ozone administration (20 and 40 ug/ml) significantly alleviated the pathological changes of intestinal tissue-induced by ARR, accompanied by the decreased loose stools rate, Bristol score and increased abdominal withdraw reflex. However, 80 ug/ml of ozone intervention played opposite roles in the aforementioned changes with 20 and 40 ug/ml of ozone. Additionally, remarkably elevated reactive oxygen species (ROS), malonaldehyde (MDA), superoxide dismutase (SOD), 5-HT, iNOS and NO levels were observed in the ozone-treated groups (20 and 40 ug/ml), while high dose of ozone drastically improved ROS, MDA, 5-HT, iNOS and NO levels but reduced the activity of SOD. Consistently, the contents of inflammatory factors were decreased after low and middle doses of ozone administration. However, high dose of ozone aggravated the inflammatory injury. Moreover, 20 and 40 ug/ml ozone upregulated TRPV1 and TRPV4 expression but downregulated 5-HTR3A expression, which was restored after 80 ug/ml of ozone intervention. Remarkably, the levels of NF-κB p65 and iNOS were dose-dependently enhanced following ozone treatment.

**Conclusions:**

Taken together, low concentration of ozone attenuated intestinal injury induced by ARR via balancing oxidative stress and inflammation, but high concentration of ozone exacerbated the intestinal injury, which might be related to the 5-HT and TRPV signaling.

## Introduction

Anterior resection syndrome (ARS) is a disease of bowel dysfunction that is common after distal colorectal resection in the treatment of colorectal cancer, accompanied by a series of symptoms of defecation dysfunction, such as frequent defecation, increased frequency of defecation, difficult urgent discharge, anal swelling, defecation incontinence, as a result of weakening the reservoir and neurosensory capacity of the rectum [1, 2]. The prevalence of ARS is high, and more than 80% of rectal malignancies individuals who undergo sphincter-sparing surgery experience varying degrees of severity [3]. Therefore, it is urgent to obtain an improved understanding regarding the pathogenesis underlying ARS to identify and develop more effective therapeutic targets for the treatment of this disease.

Ozone (O_3_) is a colorless gas, whose basic function is to protect humans from harmful effects of UV radiation. In recent years, accurate medical ozone generators have been used to investigate the mechanisms and action of ozone in clinical trials [4, 5]. Ozone treatment restores chronic oxidative stress via modulating the changed cellular redox balance [6]. Ozone therapy has become a complementary medical approach in the treatment of a wide range of diseases [7, 8]. It has been reported that therapeutic dosage of ozone can suppress chemically induced damage of nerve roots in radiculoneuritis rat [9]. Ozone therapy has a beneficial effect on anastomotic healing of the colon in the presence of peritonitis [10]. A recent study performed by the current authors indicated that ozone intervention inhibits tissue factor expression and effectively attenuates the intestinal mucosal injury in mice [11]. Therefore, whether ozone treatment can improve the ARS captures our interest in research.

In the present study, we aimed to investigate the effects of ozone treatment on ARS. An anterior rectal resection (ARR) rat model was established by low anterior resection. After different doses of ozone treated rats by enema, the therapeutic effects of ozone treatment on intestinal mucosal injury of rats with ARR were evaluated and the potential mechanisms related to 5-hydroxytryptamine (5-HT) and transient receptor potential vanilloid (TRPV) signaling were explored. Our findings might present novel insights into the mechanism of ARR physiology and new strategies for developing therapeutic interventions.

## Results

### Low dosage of ozone treatment alleviates the intestinal mucosal tissue injury in rats with anterior resection of rectum

**Fig. 1:**
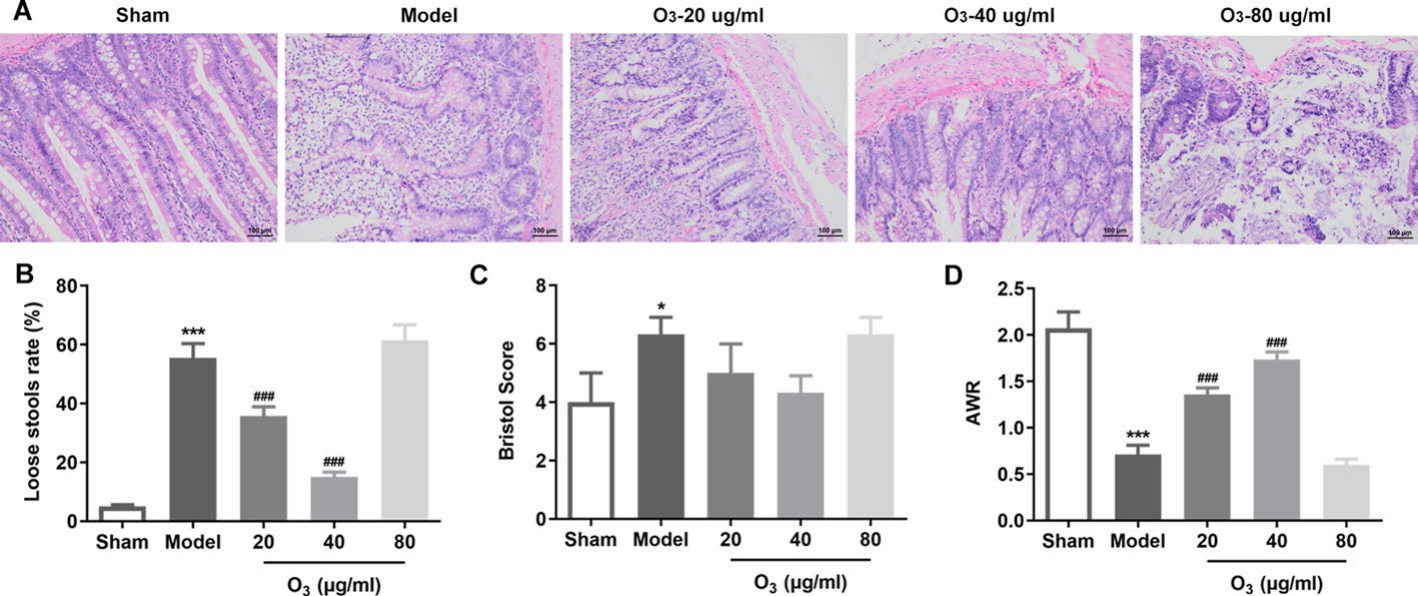
Low dosage of ozone treatment alleviated the intestinal mucosal tissue injury in rats with anterior resection of rectum. **A** The pathological changes of intestinal mucosal tissues were examined with H&E staining. **B**–**D** The rate of loose stools, Bristol stool grade and abdominal withdrawal reflex (AWR) were evaluated. ^*^P < 0.05, ^***^P < 0.001 vs. sham; ^###^P < 0.001 vs. model

To study the therapeutic effects of ozone intervention on intestinal mucosal tissue injury in rats with anterior resection of rectum, pathological changes of intestinal tissues in each group were detected using H&E staining. As displayed in Fig. 1A, the sham group rats had a normal and well-organized intestinal tissue structure. On the contrary, notably necrosis, oedema and inflammatory infiltration were observed in intestinal tissues of rats in the model group in comparison to the sham group. Treatment with ozone at the concentrations of 20 and 40 ug/ml markedly attenuated the above-mentioned intestinal tissue damage when compared to the model group, especially in the 40 ug/ml group. However, 80 ug/ml ozone enema exhibited no significant improvement in intestinal mucosal tissues compared with the model group. Additionally, the rate of loose stools and Bristol stool grade were significantly increased while the AWR was remarkably decreased in the model group compared with the sham group, which were conspicuously reversed after ozone treatment (20 and 40 ug/ml) (Fig. 1B–D). By contrast, 80 ug/ml ozone intervention presented no significant difference by statistics analysis relative to the model group. These results indicate that low dosage of ozone (20 and 40 ug/ml) enema ameliorate the intestinal tissue injury in rats with anterior resection of rectum.

### Ozone intervention affects the oxidative stress in rats with anterior resection of rectum

**Fig. 2:**
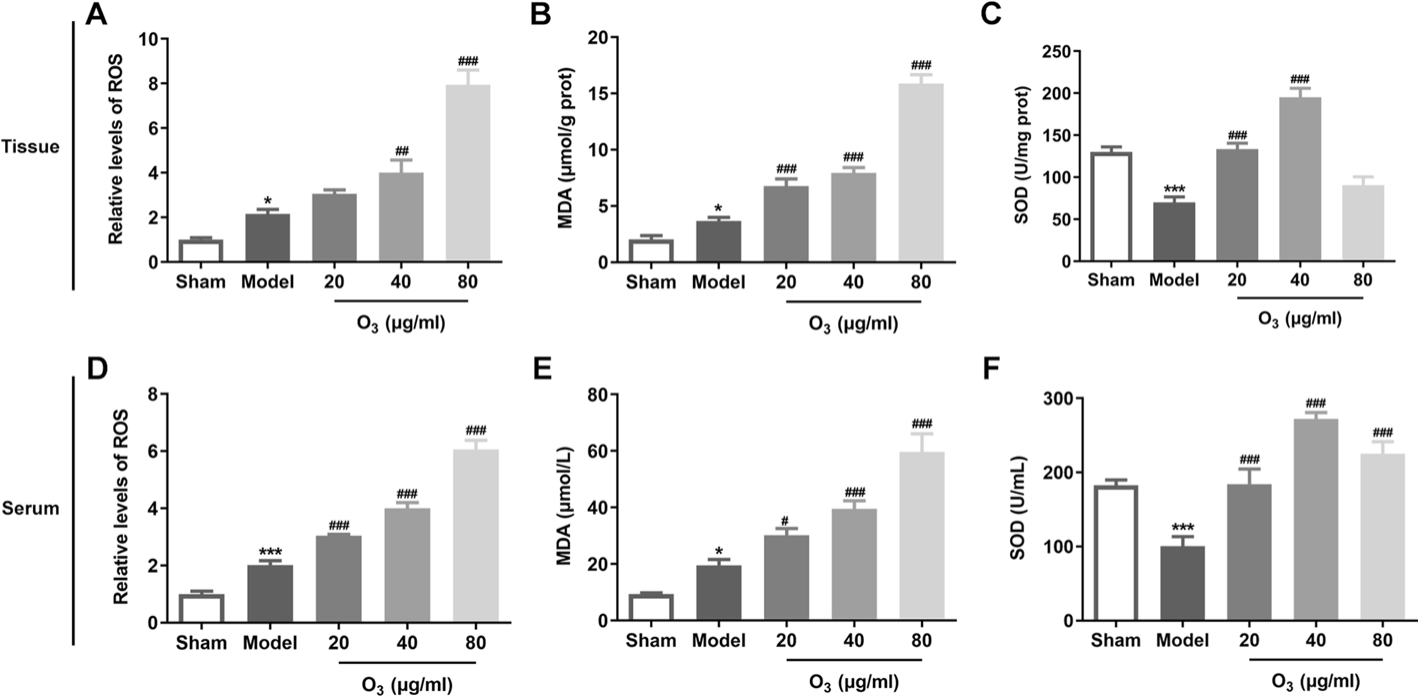
Ozone intervention affected the oxidative stress in rats with anterior resection of rectum. **A**–**C** The levels of ROS, MDA and SOD in intestinal mucosal tissues were tested by means of commercially available kits. **D**–**F** The levels of ROS, MDA and SOD in serum were tested by means of commercially available kits. ^*^P < 0.05, ^***^P < 0.001 vs. sham; ^#^P < 0.05, ^##^P < 0.01, ^###^P < 0.001 vs. model

The levels of oxidative stress-related markers in mucosal tissue homogenate supernatant and serum were determined to assess the oxidative stress status in the presence or absence of ozone in rats after anterior resection of rectum. As what is observable from Fig. 2A, B, the contents of ROS and MDA were enhanced in the model group compared with the sham group, which were further elevated after treatment with ozone at the concentrations of 20–80 ug/ml. Meanwhile, significantly reduced activity of SOD antioxidant enzyme was found in the model group, whereas 20 and 40 ug/ml ozone remarkably enhanced the activity of SOD and 80 ug/ml ozone showed no significant increase in comparison to the model group (Fig. 2C). Consistently, the levels of aforementioned markers in the serum of each group presented the same changing trends as those in the intestinal tissues (Fig. 2D–F). These data suggest that low dose of ozone (20 and 40 ug/ml) and high dose of 80 ug/ml play different roles in oxidative stress in rats with anterior resection of rectum.

### Ozone enema affects the inflammatory responses in rats with anterior resection of rectum

**Fig. 3:**
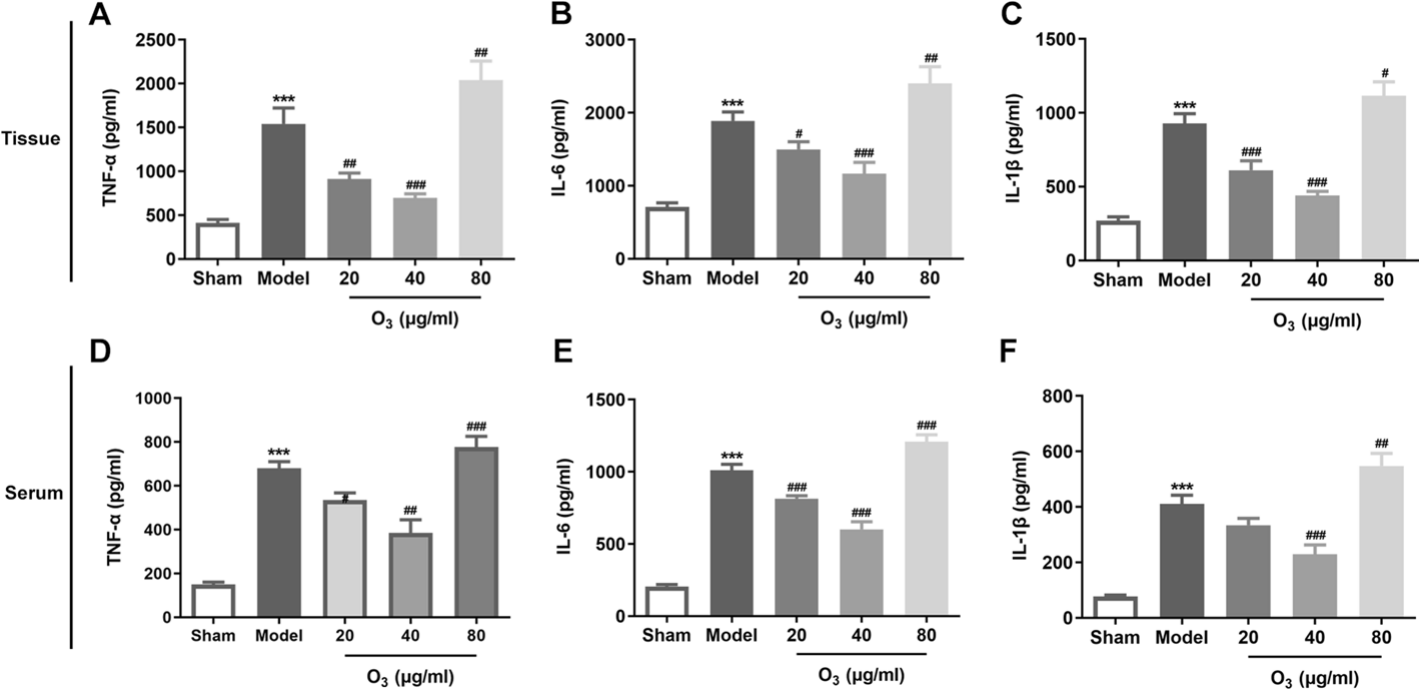
Ozone enema affected the inflammatory responses in rats with anterior resection of rectum. **A**–**C** The contents of TNF-α, IL-6 and IL-1β in intestinal mucosal tissues were evaluated with ELISA kits. **D**–**F** The concentrations of TNF-α, IL-6 and IL-1β in serum were assessed using ELISA kits. ^***^P < 0.001 vs. sham; ^#^P < 0.05, ^##^P < 0.01, ^###^P < 0.001 vs. model

It was found that anterior resection of rectum led to the significant increase of inflammatory factors including TNF-α, IL-6 and IL-1β in intestinal mucosal tissues in comparison to the sham group (Fig. 3A–C). By contrast, 20 and 40 ug/ml ozone enema notably decreased the levels of these inflammatory factors, but 80 ug/ml ozone markedly increased that of compared with the model group. Concurrently, the concentrations of these inflammatory factors in serum exhibited the same results with those in intestinal tissues (Fig. 3D–F). Through the above findings, we proved that low dose of ozone (20 and 40 ug/ml) alleviates inflammation response and high dose of ozone (80 ug/ml) exacerbates it in rats with anterior resection of rectum.

### Ozone enema regulates the expression of 5-HT in rats with anterior resection of rectum

**Fig. 4:**
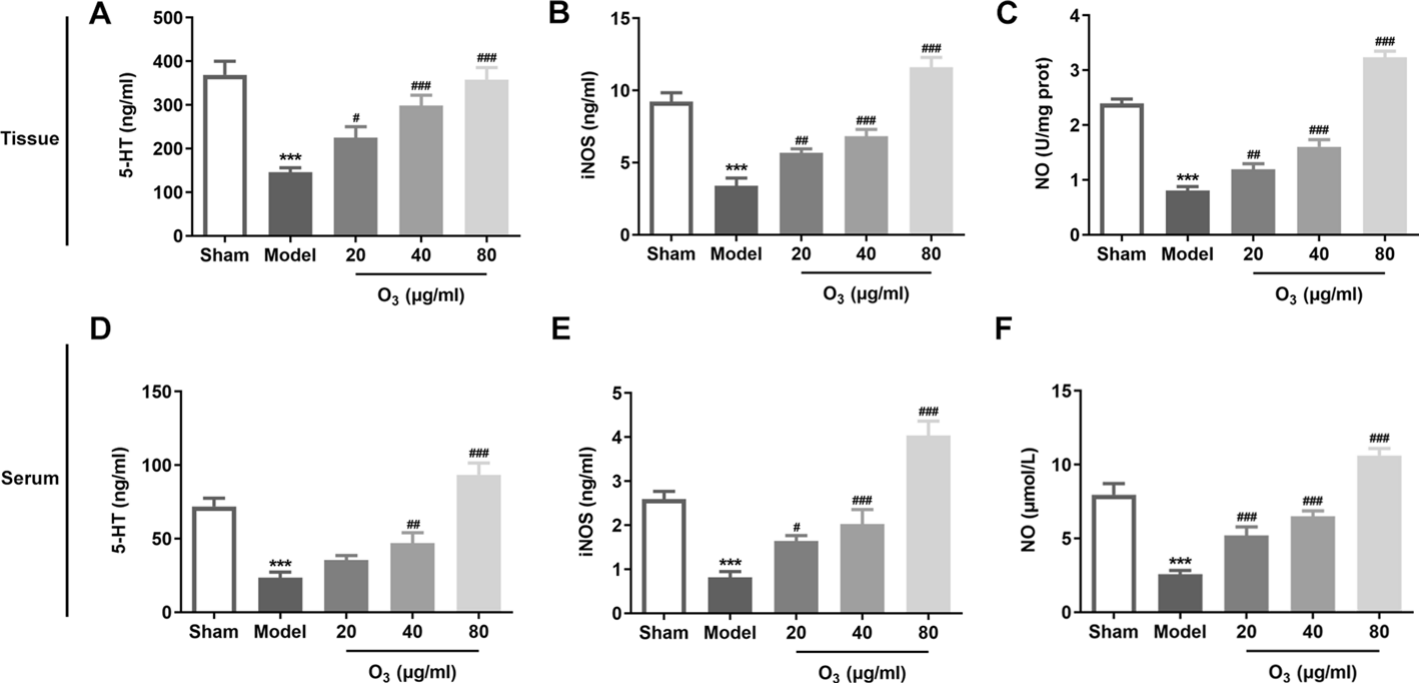
Ozone enema regulated the expression of 5-HT in rats with anterior resection of rectum. **A**–**C** The levels of 5-HT, iNOS and NO in intestinal mucosal tissues were tested by kits. **D**–**F** The levels of 5-HT, iNOS and NO in serum were tested by kits. ^***^P < 0.001 vs. sham; ^#^P < 0.05, ^##^P < 0.01, ^###^P < 0.001 vs. model

It has been reported that 5-HT is an important neurotransmitter in the regulation of bowel movement. Therefore, the level of 5-HT was determined in this study. As shown in Fig. 4A, the content of 5-HT in intestinal mucosal tissues was apparently decreased in the model group compared with the sham group. Ozone enema dose-dependently elevated 5-HT levels in comparison to the model group. Additionally, the concentrations of iNOS and NO in model group was lower than that in the control group (Fig. 4B–C). Conversely, ozone intervention conspicuously enhanced the levels of iNOS and NO in a concentration-dependent manner relative to the model group. Concurrently, in the serum, ozone enema significantly upregulated anterior resection of rectum-induced downregulation of 5-HT, iNOS and NO levels (Fig. 4D–F), which were in accordance with that in intestinal mucosal tissues. These observations reveal that ozone enema can regulate the expression of 5-HT in rats with anterior resection of rectum.

### Ozone enema modulates the TRPV1 and TRPV4 signaling in rats with anterior resection of rectum

**Fig. 5:**
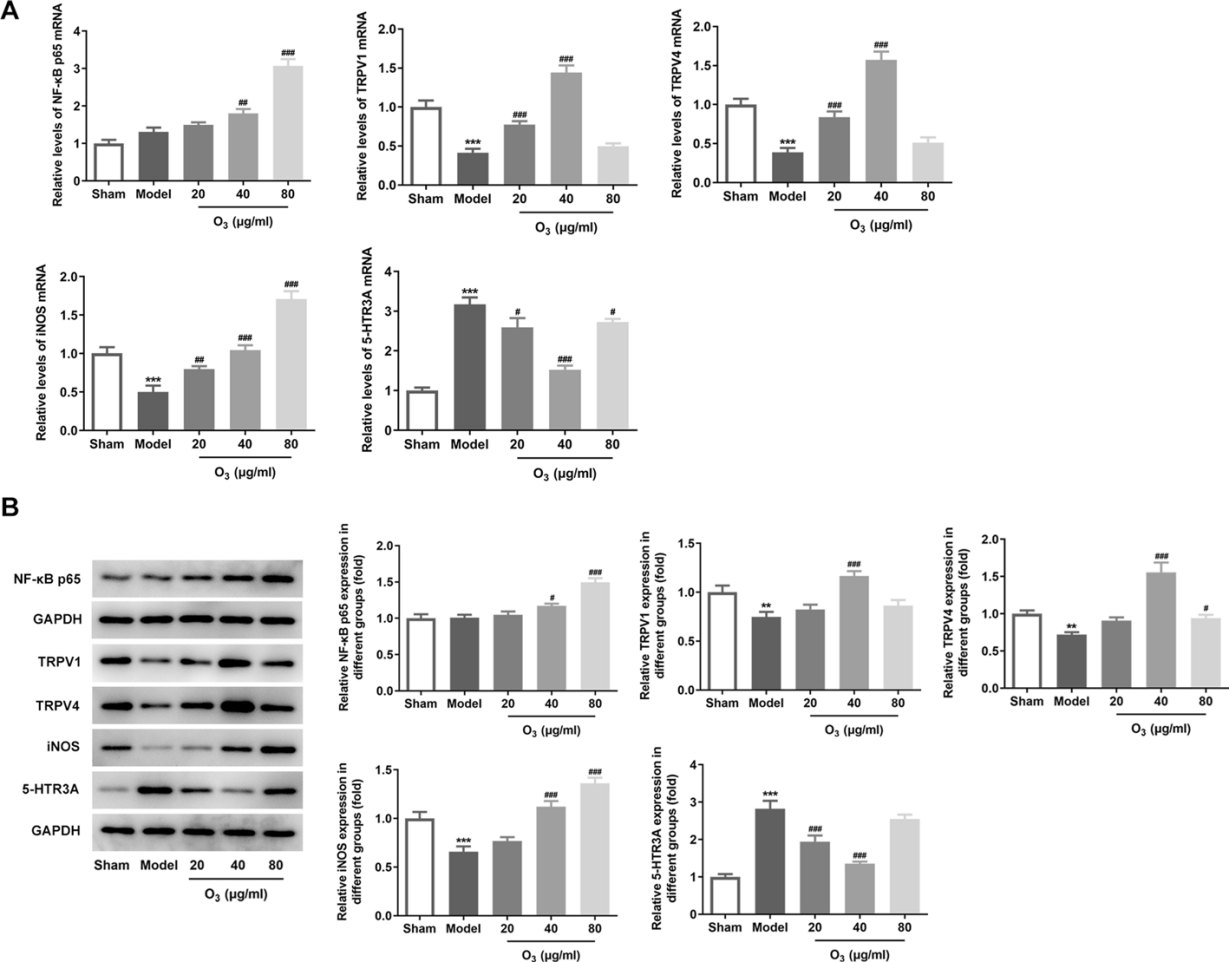
Ozone enema modulated the TRPV1 and TRPV4 signaling in rats with anterior resection of rectum. The expression of NF-κB p65, TRPV1, TRPV4, iNOS and 5-HTR3A was measured using **A** RT-qPCR and **B** western blot analysis, respectively. ^*^P < 0.05, ^**^P < 0.01, ^***^P < 0.001 vs. sham; ^#^P < 0.05, ^##^P < 0.01, ^###^P < 0.001 vs. model

As observable from Fig. 5A, B, the expression of NF-κB p65 was upregulated after anterior resection of rectum stimulation, but had no significant difference when compared with the sham group. Remarkably, ozone treatment dose-dependently elevated NF-κB p65 expression in comparison to the model group. Simultaneously, anterior resection of rectum led to significant decrease in the levels of TRPV1, TRPV4 and iNOS expression, which was restored by ozone enema with the dosage of 20 and 40 ug/ml. By contrast, 80 ug/ml ozone enema had no impact on the expression of TRPV1 and TRPV4, but had unregulated iNOS expression relative to the model group. Moreover, notably increased 5-HTR3A level was observed in the model group, which was decreased after ozone enema. Overall, these data suggest that ozone enema modulated the TRPV1 and TRPV4 signaling in rats with anterior resection of rectum.

## Discussion

With the standardized application of total mesorectal excision and neoadjuvant therapy in surgical treatment of rectal cancer, as well as the application of various minimally invasive techniques, the 5-year survival rate of patients with rectal cancer increases gradually. ARS is considered to be the main cause of the decline in quality of life caused by the dysfunction of the colorectal and defecation after rectal resection. In the present study, we demonstrated that low dose of ozone treatment could attenuate intestinal injury induced by ARR via balancing oxidative stress and inflammation, but high concentration of ozone exacerbated the intestinal injury, which might be related to the 5-HT and TRPV signaling.

It has been reported that ozone treatment can relieve oxidative stress via regulation of the changed cellular redox balance, thereby normalizes organic peroxide levels and activates the antioxidant system [12]. In recent years, ozone therapy has been widely applied to the treatment of multiple human diseases as a complementary medical approach [8]. It has been reported that preconditioning with small doses of ozone protects kidney, intestinal mucosa and brain from ischemia–reperfusion injury in animal models [13–15]. Wu et al. [9] demonstrated that therapeutic dosage of ozone can attenuate chemically induced damage of nerve roots in radiculoneuritis rat. In rat model of experimental uveitis, ozone therapy decreases inflammation in histopathologic examination when compared with the sham group [16]. Ozone therapy has been proven effective in the treatment of experimental model of rheumatoid arthritis [17]. Additionally, ozone therapy contributes to the recovery of testicular damage in an experimental model of testicular torsion in rats by reducing oxidative stress [18]. Importantly, emerging evidence supports the notion that ozone administration inhibits tissue factor expression and exhibits beneficial effects on intestinal mucosal injury in mice [11]. In the present study, the effects of ozone treatment on low anterior resection-induced rat model were investigated for the first time, and we found that low concentration of ozone (20 and 40 ug/ml) notably alleviated intestinal injury via balancing oxidative stress and inflammation, but high concentration of ozone (80 ug/ml) aggravated the intestinal injury.

Ozone acts as a bio-regulator by inducing the release of endothelial cell factors and by normalizing cellular redox balance when it comes in contact with a biological fluid [19]. Research has proposed that the expression of 5-HT is significantly reduced in patients with anterior rectal resection, which may be related to the pathogenesis of ARS [20]. Serotonin, a 5-HT3 receptor antagonist, has been proved to relieve the symptoms of low anterior resection syndrome [21]. The role of 5-HT in the intestinal tract is generally considered as a neurotransmitter that promotes intestine to function normally, and it is also one of the signal sources of TRPV function (a proton and heat-gated channel) mediated in the intestinal lumen [22]. TRPV is present in visceral afferent neurons, and TRPV1 and TRPV4 can affect transcription factors and lead to the standardization of cell functions by affecting the intracellular calcium ion flow [23]. INOS and NO are also very important transmitters in the intestine, and elevated iNOS content will cause an increase in the content of NO, leading to smooth muscle relaxation [24]. Meanwhile, NF-κB is one of well-known iNOS inducers, that is largely expressed in inflammatory conditions [25]. In the present study, ozone treatment (20 and 40 ug/ml) upregulated the expression of 5-HT, TRPV1, TRPV4, NF-κB, iNOS and NO, but downregulated that of 5-HTR3A expression. However, high dose of ozone intervention reduced the levels of TRPV1, TRPV4, but enhanced that of 5-HT, NF-κB, iNOS, NO and 5-HTR3A. These findings suggest that ozone enema could regulate the 5-HT and TRPV signaling in rats with anterior resection of rectum.

## Conclusion

In summary, the present study demonstrated that low concentration of ozone attenuated intestinal injury induced by ARR via balancing oxidative stress and inflammation, but high concentration of ozone exacerbated the intestinal injury, which might be related to the 5-HT and TRPV signaling. This study is the first to investigate the effects of ozone treatment on intestinal mucosal injury of rats with anterior resection of rectum and to clarify the underlying regulatory mechanisms during its process. Our findings present novel insights into the mechanism of ARR physiology and new strategies for developing therapeutic interventions. Various functional phenotypes of intestinal tissues after ozone administration and the underlying mechanisms related to mitochondrial apoptosis, autophagy or endoplasmic reticulum stress will be investigated in the following experiments.

## Materials and methods

### Animals

A total of 30 specific pathogen-free (SPF)-grade male Sprague-Dawley (SD) rats (200–250 g) were obtained from Shanghai Family Planning Research Institute (Shanghai, China). All rats were individually in a specific pathogen-free facility (22 ± 2 °C) under standard conditions with 12 h light/dark cycle. Standard rat food and tap water were available. All animal experiments in the study were maintained and used according to the protocols approved by the Animal Experiment Ethics Committee of Nanjing University of Chinese Medicine.

### Model establishment and ozone treatment

SD rats were randomly assigned into five groups (N = 6 in each group) as follow: sham, model, O_3_-20 ug/ml, O_3_-40 ug/ml and O_3_-80 ug/ml. Colon tissues about 5 cm from the anus of the rats were taken from animals in the model and O_3_-treated groups were removed and sutured. 24 h after model establishment, different doses of ozone (prepared by Germany Carter's original medical ozone instrument connecting with oxygen tank) were employed to treat rats by enema daily for five consecutive days. Rats after the last administration were fasted for food for 24 h, the rats were anesthetized with intraperitoneal injection of 3% pentobarbital sodium. Intestinal tissue and blood samples were obtained for the following experiments.

### Measurement of the rate of loose stools

The loose stool rate of rats in each group was observed 6 h after the last enema. Rat in each group was placed separately in a cage with a stainless steel grid at the bottom. The trays under the steel grid were lined with filter paper to observe rat droppings. The rate of loose stools was calculated as follows: the number of loose stools/total number of bowel movements × 100%.

### Abdominal withdraw reflex (AWR) test

The AWR test was performed after rats being fasted for food for 24 h. Rats were anesthetized with intraperitoneal injection with 3% pentobarbital sodium, the balloon end of the double-channel catheter (diameter 2.7 mm) coated with paraffin oil was inserted about 2 cm from the anal margin, and the catheter was fixed at the root of the rat tail with adhesive tape. The rats were put into a special transparent observation box, in which they could not turn around but could only move forward and backward. After adaptation for 30 min, inflating the balloon with capacity of 1.0, 1.5, 2.0 mL was, respectively, used to enlarge the gut at an interval of 10 min each time. The procedure was repeated for three times for each rat, and the average value of the three times was taken. The scoring standard was conducted according to the previous study [26].

### Bristol stool scale

Bristol stool grading was performed 4 h after the last enema. The feces of the rats for 4 h were collected, weighed (wet weight), dried, and weighed again (dry weight). Fecal water content (%) = (wet weight − dry weight)/wet weight × 100%.

### Histological examination

Appropriate weight intestinal tissues were soaked in 4% paraformaldehyde overnight. After dehydration in gradient ethanol, the tissues were made transparent with xylene, and the blocks were embedded in paraffin. The tissue samples were subsequently embedded in paraffin and cut into 5-μm-thick sections, which were then deparaffinized in xylene and rehydrated in a descending ethanol series. After being dehydrated with graded ethanol and xylene, the sections were stained with hematoxylin and eosin (H&E) using standard techniques. The stained slides were observed under a light microscope (Olympus Corporation, magnification, × 200).

### Measurement of oxidative stress-related markers

Intestinal tissues were cut into small pieces, which were homogenized with RIPA lysis buffer (Absin, Shanghai, China), centrifuged at 12,000*g* for 10 min, and the supernatant was collected. The blood sample was centrifuged at 12,000*g* for 10 min to obtain serum.

The contents of reactive oxygen species (ROS) and malondialdehyde (MDA) as well as the activity of superoxide dismutase (SOD) in tissue homogenate supernatant or serum were evaluated by means of commercial kits in accordance with the specification provided by the supplier (Nanjing Jiancheng Bioengineering Institute; Nanjing, China).

### Test for inflammatory factors

Enzyme-linked immunosorbent assay (ELISA) kits were adopted for detection of the levels of tumor necrosis factor-alpha (TNF-α), interleukin (IL)-6 and IL-1β in tissue homogenate supernatant or serum following the manufacturer’s protocols (Shanghai Xitang Biotechnology Co., Ltd., Shanghai, China). The optical density values were read on a plate reader (BioTek Instruments, Winooski, VT, USA).

### Evaluation of 5-hydroxytryptamine (5-HT), inducible nitric oxide synthase (iNOS) and nitric oxide (NO)

The levels of 5-HT, iNOS and NO in tissue homogenate supernatant or serum was determined with commercial kits according to the specification provided by the supplier (Nanjing Jiancheng Bioengineering Institute; Nanjing, China).

### Reverse transcription-quantitative PCR (RT-qPCR)

Total RNA was extracted from tissues using TRIzol® reagent (Thermo Fisher Scientific, Inc.). Total RNA was reverse transcribed into cDNA using the First Strand cDNA synthesis kit (Thermo Fisher Scientific, Inc.), according to the manufacturer’s protocol. qPCR was subsequently performed with 2 μg cDNA using the SYBR Premix Ex Taq (Takara Bio, Inc.) and ABI 7500 equipment (Applied Biosystems; Thermo Fisher Scientific, Inc.). The following thermocycling conditions were used: Initial denaturation at 95 °C for 10 min; followed by 40 cycles of denaturation at 95 °C for 15 s and annealing at 60 °C for 1 min; and a final extension of 10 min at 72 °C. Glyceraldehyde-3-phosphatedehydrogenase (GAPDH) was chosen as the reference gene for normalization. The 2^−ΔΔCq^ method was used to compare relative expression levels [27].

### Western blot analysis

For immunoblotting, the intestinal tissues were lysed on ice and centrifuged at 4 °C and at 12,000 g for 10 min. The supernatant was extracted using RIPA buffer (Absin, Shanghai, China) containing proteinase inhibitor cocktail (Innovation, USA). The concentration of protein was then determined using a BCA kit (Beyotime Institute of Biotechnology). 40 μg protein/lane was separated by 10% SDS-PAGE. The separated proteins were subsequently transferred onto polyvinylidene fluoride membranes (EMD Millipore) and blocked with 5% skimmed milk for 1.5 h. The membrane was incubated with anti-TRPV1 (Proteintech, Chicago, USA), anti-TRPV4 (Abcam, Cambridge, UK), anti-NF-κB p65 (Bioss, Beijing, China), anti-5-HTR3A (Bioss, Beijing, China) and anti-iNOS (Bioss, Beijing, China) antibodies overnight at 4 °C, followed by incubation with secondary antibodies (Abcam, Cambridge, MA, USA) for 1 h at room temperature. Protein bands were scanned and visualized using an enhanced chemiluminescence detection system (EMD Millipore). Each band was quantified via Image J software (National Institutes of Health). The gray value of the target protein was normalized to that of GAPDH.

### Statistical analysis

All experiments were performed in triplicate. The results are presented as the means ± standard deviation and statistical analysis was conducted with the GraphPad Prism 8.0 software. One-way ANOVA followed by Tukey’s post hoc test was used to compare differences among multiple groups. P value less than 0.05 stands for significant difference.

## Data Availability

The datasets used and/or analyzed during the present study are available from the corresponding author on reasonable request.
